# Reflecting on Peer Feedback in Problem-Based Learning: Implementing a Group Function Tool

**DOI:** 10.7759/cureus.75027

**Published:** 2024-12-03

**Authors:** Matthew Mellon, Hanna Van Dierdonck, Leo Morjaria, Keyna Bracken, Matthew Sibbald

**Affiliations:** 1 Medicine, McMaster University, Hamilton, CAN; 2 Family Medicine, McMaster University, Hamilton, CAN; 3 McMaster Education Research, Innovation, and Theory (MERIT) Program, McMaster University, Hamilton, CAN; 4 Cardiology, McMaster University, Hamilton, CAN

**Keywords:** group function, health-professions education, learning tool, peer feedback, problem-based learning

## Abstract

Introduction

Self-directed peer feedback is integral to the problem-based learning (PBL) process, but poorly scaffolded feedback processes can be inefficient and ineffective and there is little guidance on how students should structure these processes. This study aims to identify implementation considerations for a group function reflection tool and explore group feedback behaviours around the operationalization of the tool.

Methods

We conducted a qualitative study informed by direct content analysis using the group function reflection tool and conducted semi-structured focus groups in 2024 with 24 medical students and two tutors participating in a PBL curriculum. Students conducted peer feedback using the tool over four weeks, submitted feedback through an online form, and reflected on their experiences in focus groups. We analyzed feedback responses and transcripts in a staged approach, sensitized by three frameworks: the Human Factors Framework, the Task-Gap-Action model of feedback, and *Thanks for the Feedback:* Appreciation, Coaching, and Evaluation.

Results

We constructed five themes: 1) appreciative feedback is often under-valued, 2) there is tension between structure and flexibility in the feedback process, 3) the interplay between written and verbal feedback, 4) the density of feedback requires careful optimization, and 5) the tool as a threat to tutors.

Discussion

Operationalization of the tool exposed tensions around the peer feedback process. The tool reinforced the importance of a self-guided process for peer feedback which also requires prompting. It raised assumptions about the PBL feedback process which should be further studied to better understand peer feedback in broader contexts.

## Introduction

Problem-based learning (PBL) has become a widely used pedagogical model in health professions education due to its small group format which is meant to model the collaborative and dynamic nature of healthcare teams [1,2]. Although its primary purpose is to facilitate knowledge acquisition through problem-solving, peer feedback is an equally integral part of the process [3]. Indeed, the peer feedback process has been identified as a crucial modelling opportunity for the skills required in healthcare practices [3]. Many PBL curricula encourage and incorporate peer feedback in a variety of formats to facilitate the acquisition of collaboration and teamwork skills that are relevant in the workplace [4]. Peer feedback can range from informal point-in-time advice to formal structured discussions, usually tutor-facilitated, at crucial points in the PBL process (end-of-tutorial or mid-unit feedback) [5].

Some theoretical frameworks are relevant to understand the nature of peer feedback in the PBL context. The Task-Gap-Action Model of Feedback outlines key elements of peer feedback that proceed in a stepwise process commencing with feedback around the task (identification of the event on which feedback is provided), gap (acknowledgement of a gap between performance and an ideal standard), and action (using the feedback to develop a plan) [6]. Each step requires deeper reflection than the previous one. Thanks for the Feedback: Appreciation, Coaching, and Evaluation describes three types of peer feedback: appreciation (identification of positive group behaviours to continue), coaching (providing suggestions to improve group function), and evaluation (an appraisal of group behaviours warranting modification) [7].

Peer feedback has become an essential part of the PBL process largely due to its proven ability to enhance outcomes. Student perceptions of peer assessment in non-PBL academic contexts are generally positive; peer assessment increases students’ commitment to their learning and sense of responsibility for their peers’ learning [8]. Similarly, peer feedback in PBL has been shown to enhance student contributions to the group and to improve academic outcomes, especially for initially low-performing students [5,9,10]. PBL peer feedback has also been linked to the development of enhanced self-reflection and professionalism skills in medical learners [3,11].

However, students encounter difficulties with the overly subjective nature of peer feedback [4,12]. They are often unaware of the attributes of well-functioning groups and struggle to guide feedback discussions accordingly [13]. Students also report that feedback often does not translate to improvements in group function [14]. Peer feedback given in non-optimal circumstances, poor timing or inadequate social dynamics, often falls through prior to implementation, and it may take several failed attempts at feedback to give rise to meaningful changes [14-16]. Existing literature highlights the need for teaching around peer feedback [4,13,17,18].

Scaffolding of the peer feedback process has been shown to be useful when it provides an adequate level of guidance that preserves the self-directed nature of the feedback process [18-20]. Here, scaffolding refers to the provision of loose structure to learning processes, which may be gradually removed as students develop their own processes. The recent development of a PBL group function reflection tool presents an opportunity to scaffold the feedback process while preserving group autonomy [21]. Li et al. propose a framework specific to the PBL context that identifies common topics of group function feedback given by students, which fall under four categorical domains [21]. Given the relationship between strong group function and outcomes, the tool appropriately encourages students to reflect and improve upon group function [10]. The framework was developed through a scoping review of the feedback literature and consolidation of existing themes into broad categories. The framework doubles as a guide to focus students on effective group behaviours and as a tool to streamline the feedback process.

However, uncertainties around the tool’s implementation emerged during its development. The authors discussed student concerns around time constraints and allocating more time for feedback at the expense of discussions around content learning [21]. They also highlighted the social hesitation to give constructive feedback and how this disproportionately burdens the tutor to initiate and provide feedback [21]. Accordingly, existing literature around scaffolds in problem-based learning highlighted challenges around implementation, including motivation to use, integration with existing course organization, design, content, and unfavourable conditions for reflection [20]. Hence, further investigation is required to better understand how such scaffolds can be applied to the PBL context in a manner that enhances peer feedback, ensures their usage, and protects the self-directed nature of PBL. This study aims to identify implementation considerations for the group function reflection tool and explore group behaviours around the operationalization of the tool.

## Materials and methods

Using an electronic adaptation of the group function reflection tool, we conducted a qualitative study whereby the tool was implemented among medical students enrolled in a PBL curriculum. Combining written feedback collection with semi-structured focus group discussions, we used direct content analysis to assess implementation, feedback behaviour around the tool, and considerations for its future implementation.

Study team

The study team was selected to promote diversity of perspectives and experiences. MM is a medical student with experience in educational development and as a student in a PBL curriculum. HVD and LM are medical students with experience in qualitative research and as students in a PBL curriculum. KB is a clinician scientist with experience as an educator, tutor, and administrator of a PBL curriculum. MS is a clinician scientist with experience in PBL as a tutor, educator, and researcher. KB and MS are also the Pre-Clerkship Director and the Associate Dean (Undergraduate) for the School of Medicine, respectively. They had no direct involvement with participants to mitigate potential impact of their roles on data collection.

Context and participants

**Figure 1:**
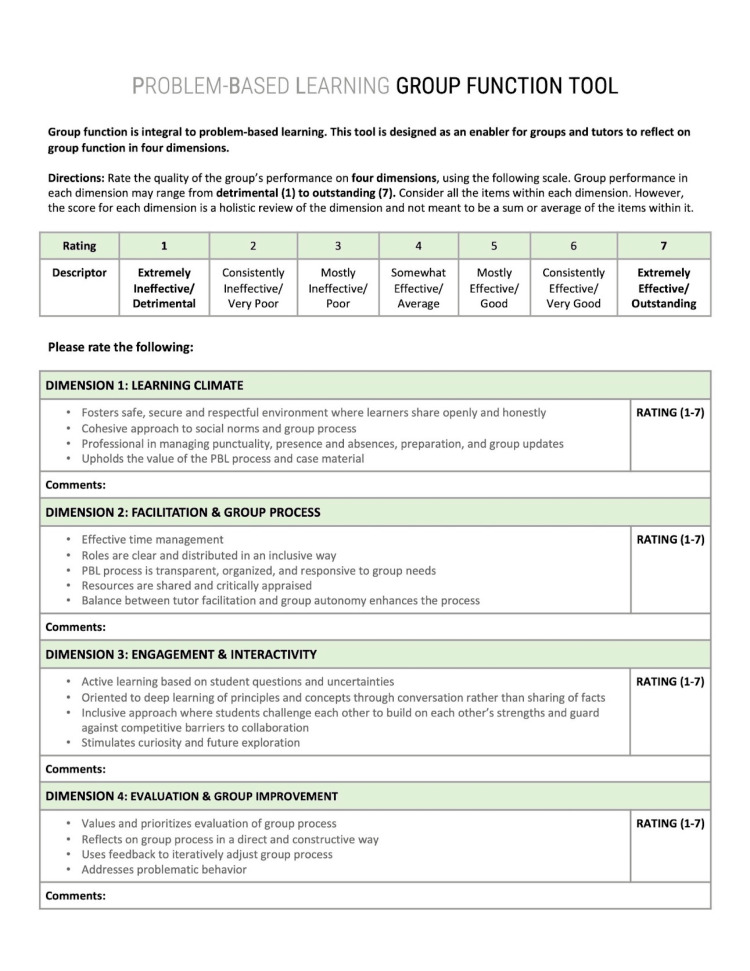
Problem-based learning group function reflection tool Permission has been obtained to reproduce this image from the original author [21].

We conducted this study at the Michael G. DeGroote School of Medicine at McMaster University, where PBL is the foundational pedagogy in the undergraduate medical curriculum. The pre-clerkship curriculum is divided into five Medical Foundations (MF), each eight to 12 weeks long with two PBL tutorials each week. Groups typically contain eight students and one tutor. Groups dedicate five to 10 minutes at the end of each tutorial session for peer feedback. The feedback process is self-directed by group members, and formal teaching around feedback is not part of the curriculum. The group function reflection tool was developed at our institution in a literature-informed iterative process described elsewhere [10,21]. It has not previously been tested by PBL groups. For this study, the tool was integrated into groups’ existing feedback processes and used to augment feedback that they would normally conduct. The tool is shown in Figure 1.

We used a purposive recruitment strategy aimed at tutors who were facilitating groups of first-year students in their third MF (MF3). Inclusion criteria for student participants were (i) to be an undergraduate MD student enrolled at McMaster University participating in MF3 at the time of the study and (ii) have at least four of their group members also participating. Inclusion criteria for tutors were (i) to be an active tutor in the MD program at McMaster University in MF3 at the time of the study and (ii) have at least five of their student group members participating in the study. An open call for participation was directed to MF3 tutors. Tutors obtained verbal consent from students in their groups before proceeding with the study. Recruitment was conducted via tutors to streamline the process and increase the likelihood of entire groups participating together. Students also provided passive consent upon submission of the initial feedback forms, then signed consent forms prior to participation in focus groups. Tutors were invited to participate in a separate focus group at the conclusion of the study and signed consent forms prior to participation.

Data collection

We collected data from participants over four weeks during the first half of MF3, between January and February 2024. Participating groups were given a Google Forms®-based questionnaire (Mountain View, CA) modelled after the group function reflection tool. The form utilized the Autocrat function which would automatically generate a document with the group’s consolidated feedback and email it to a chosen group member in real-time upon submission. Groups were provided with an information sheet which explained how to use the Google Form and provided instructions on how to conduct feedback. The information sheet suggested that group members fill out the form individually, use it to frame a subsequent group discussion, and record a summary of the discussion on the same document. Groups were still encouraged to direct their own feedback process. This process enabled the research team to maintain a written record of the group’s feedback. Groups used this feedback form a total of four times, once per week for four weeks, beginning the second week of MF3. We chose this timeline based on evidence that peer feedback and group development in experienced PBL groups is richest near the beginning of a group’s lifespan [14].

We conducted semi-structured focus group discussions with participating groups during the subsequent two weeks of MF3, based on group availability. Focus groups were 30-40 minutes and were conducted during each group’s scheduled tutorial. We chose this timeline to give enough time for feedback implementation, while ensuring sufficient recency for recall and reporting. Participants were provided with a record of their feedback beforehand, then asked to reflect on and discuss their impressions of the tool, their feedback processes, and factors influencing the implementation of their feedback. An additional focus group was scheduled for participating tutors to discuss their impressions of the tool and the feedback process. Focus group discussions were audio recorded through Zoom® (San Jose, CA) and transcribed verbatim. Transcriptions were generated by a secure external service, Scribie® (San Francisco, CA). Transcripts were de-identified by the research team, and focus group recordings were deleted.

Data analysis

We reviewed the feedback form responses and transcripts in a staged approach moving from qualitative description to direct content analysis [22]. Initial readings relied on a mixed approach of inductive and deductive coding to identify key themes using annotations of shared online documents [22,23]. Coding was sensitized by three theoretical frameworks in a manner consistent with direct content analysis:

1) The Human Factors Framework emerged from a review to identify human factors and their interactions with automated applications [24]. The investigators identified four factors to consider when designing automated systems to be functional for their users: perception, interface usability, cognitive workload, and trust. We see these factors as essential to the validity and user satisfaction of our automated feedback form. As such, we used this framework in a deductive manner to assess participants’ perceptions of the feedback form and their impressions of how it performs on the four factors.

2) The Task-Gap-Action Model was initially used for qualitative description in a deductive manner, but it did not contribute substantially to thematic analysis [6].

3) Thanks for the Feedback: Appreciation, Coaching, and Evaluation was identified in an inductive manner following our initial data review and found it to better classify the content of peer feedback as compared with the Task-Gap-Action model [7].

All members of our team discussed codes and linkages in regular meetings, with subsequent transfer of the group’s coding into qualitative analysis software, Dedoose® (Manhattan Beach, CA). This enabled an iterative transition to axial coding identifying more in-depth linkages between material. We continued this process until saturation was achieved, defined as coherence and empiric support for themes in line with our research objectives and with meaningful impact for PBL educators and students [25].

Approach to methodological rigour

We adopted a systematic approach to rigour through data collection and analysis. Focus groups opened with a structured pre-briefing to establish expectations around confidentiality and psychological safety for the focus group process. Transcripts were verified for accuracy and anonymity prior to analysis. The analytic team was constructed to balance perspectives including academics, leaders, and students with perspective into the PBL process. This enabled deeper analysis of implementation considerations by incorporating perspectives of all stakeholders in the tool’s usage. Analysis involved triangulating data across focus groups, examining multiple theoretical perspectives, and revisiting codes and themes in the analytic process. Reflexive practices were employed to acknowledge and neutralize researcher bias while leveraging individual subjectivity such that it gave rise to unique perspectives in data analysis [26]. Our team undertook reflexive journaling to identify emergent insights and wrote memos on shared focus group transcripts. The research team met to discuss data impressions and engaged in structured team reflexive discussions. Data impressions and emerging themes were circulated and reviewed by all team members at three stages before determining saturation had been reached.

## Results

A total of three PBL groups were recruited, containing 24 participating students. All members of each group participated. All participants were first-year medical students, with 13 males and 11 females. Feedback was collected from each group on four occasions for a total of 12 feedback collection points. All students submitted responses to the feedback form at each iteration for a total of 288 responses. Two tutors participated in the tutor focus group. We identified five themes: 1) appreciative feedback is often under-valued, 2) there is tension between structure and flexibility in the feedback process, 3) the interplay between written and verbal feedback, 4) the density of feedback requires careful optimization, and 5) the tool can be perceived as a threat to tutors.

Appreciative feedback is often under-valued

Groups surprisingly provided a large volume of appreciative feedback in their responses to the feedback form compared to the amount of constructive feedback. Our intended approach to analyzing feedback was to assess congruence with the Task-Gap-Action model and understand whether the tool could encourage groups to translate feedback discussions to actionable goals [6]. However, the categorical divide of Appreciative-Coaching-Evaluation feedback was discovered to be more relevant [7]. The Task-Gap-Action paradigm is one of coaching-based feedback. While coaching was present, we were surprised to see that appreciation was far more prominent. The reasons for its over-representation were unexpected; appreciative feedback was usually not purposeful or intrinsically motivated. Rather one participant reported that they simply defaulted to appreciation in the absence of meaningful coaching feedback:

“The problem with constructive feedback for me is that…I'm personally kind of reticent to give feedback…that's negative without having suggestion of what we can do better.” (Group A)

Some participants cited avoidance of coaching and evaluation due to social discomfort, while others cited the effortful nature of constructive feedback driving them towards appreciation, which they perceived as less effortful. Participants also expressed neutral or negative attitudes towards appreciative feedback:

“Despite the fact that we didn't have a ton of negative feedback, at least in my experience, this feedback had more substance to it than any of the previous MFs…previous MFs where we sit in person, it's typically like, ‘it was so great today, guys really awesome stuff. Everyone's doing a great job.’ And that was really it.” (Group A)

This participant expressed surprise to see that feedback could be substantive despite it not being negative. They also expressed disappointment in previous feedback experiences, saying ‘that was really it’ in response to standalone appreciative feedback. In contrast, participants still indicated positive perceptions of appreciative feedback:

“I do see the value of doing the check in, just about the things that are going well and just like okay, people do generally feel this is a safe space. That's good and I think that was valuable and it was kind of nice to hear.” (Group B)

Evidently, the tool facilitates additional appreciative feedback, and participants feel that it enhanced their feedback process and group function. This further complicates the perception of appreciative feedback; participants viewed it as an unexpected asset but also as a runner-up to constructive feedback. Although groups underestimated the value of appreciative feedback and utilize it in an unintentional manner, they showed that it is still uses to derive meaningful outcomes.

There is tension between structure and flexibility in the feedback process

By nature, the tool imposes a more structured process than conventional self-directed peer feedback. Participants expressed contradictory sentiments towards the imposition of a prescribed process. They inferred strict application of the tool and sought clear guidance on how it should be implemented, but they also rejected its rigidity and craved flexibility. This posed a threat to the usability of the tool, as per the Human Factors Framework. One participant indicated that the tool’s structure had negative implications on the feedback process:

“I think it introduced a little bit of rigidity, which was, I think it made it a little bit more challenging to make it through feedback.” (Group C)

Indeed, the variable use of the Action Items portion of the tool exemplifies participants’ desire for flexibility. While one group used this section as intended, another group expressed frustration that it was forced to the end of the feedback process instead of being interspersed. The final group simply abandoned it in favour of a verbal discussion. In contrast, other participants expressed positive impressions of a more structured process:

“I appreciated that there was consistency, (be)cause it's sometimes very easy to get to the end of tutorial and have three minutes left, and then be, uh-oh we didn't give you feedback let's just do it next time, but the fact that we were externally accountable…to give feedback…that was cool.” (Group B)

Many participants also sought additional clarifications beyond what was provided on the information sheet, despite the sheet being emphasized as a suggestion rather than a rigid guide. The research team was contacted by participants with questions around timing, appropriate depth of written feedback, and the order of events in the use of the tool.

Written and verbal feedback require balance

Participants’ struggle between structure and flexibility was further emphasized by their uncertainty around whether to prioritize written feedback (directly from the tool) or verbal feedback (generated in discussions around the tool’s output). In favour of using the tool for written feedback, one participant indicated deeper self-reflection:

“I feel like in the past…when I've given feedback, it's just been me thinking on the fly. So I feel like (writing it) challenged me to think about, okay, like what is it in particular that I'd like to say about this.” (Group C)

In contrast, another participant felt that the tool was an unnecessary step on the way to more meaningful verbal feedback:

“The form is functional when the group is dysfunctional. So if the group can't have an open and honest conversation, the form will work. If the group is able to have an open and honest conversation, the form is an extra step that will just take time. We'll put in a number and answer whatever question so you can click next and then we'll have a real feedback discussion.” (Group B)

Table 1 summarizes additional benefits to written and verbal feedback expressed by participants. Although participants rejected a rigid format for written comments while also requesting a prescribed division of written and verbal feedback, a balance is most likely to be effective. The extent to which the tool dominates the feedback discussion is group- and context-dependent.

**Table 1:** Benefits of written and verbal feedback

Written Feedback	Verbal Feedback
Anonymity and safety	Greater collaboration on action plans
Accountability to engage in feedback	Additional context on feedback items
Accountability to implement action plans	Enhanced group cohesion
Opportunity for deeper self-reflection	Avoids “the job is done” attitude of written feedback
More detailed feedback content	

The density of feedback requires careful optimization

Participants cited challenges around the volume, frequency, and time dedicated to feedback when using the tool. As independent variables taken together, they constitute the density of feedback. In terms of the Human Factors Framework, these are encompassed by the workload of the tool. However, participants expressed contradictory impressions of what constituted appropriate levels of volume, frequency, and time. For the tool to function effectively, it evidently requires careful tailoring of these variables to the needs of each group. One participant acknowledged the additional time burden of generating meaningful feedback in the context of the tool:

“Having that almost forced time to sit and everyone write a little bit of feedback and then we go through it together was really helpful (compared to) past groups where kind of you get to your little five minutes of feedback at the end and everyone's just kind of like, yep, it's good.” (Group A)

In contrast, another participant expressed a negative perception of the time burden:

“I think on the…days we weren't filling out the form, we were using time more for learning and less for feedback.” (Group A)

They interestingly pit learning and feedback against each other, implying that increasing the time for feedback necessitates a trade-off with learning. This exemplifies a belief held by participants that there is a limit to the time spent on feedback. Participants also highlighted the burden of a high volume of feedback, where too much feedback at once became too overwhelming to operationalize. The tool appeared to worsen this problem given the depth and breadth of topics listed on the tool. One participant expressed these difficulties with the volume of feedback. Again, implicit to their comment was an underlying belief that there is a limit to the time spent on feedback:

“One other thing that could help with time is instead of having all four every time, maybe focus, this week we're focusing on feedback process. Let's give feedback about this. Let's focus on one section, because they did tend to run long.” (Group A)

The tool may threaten tutors

Tutor participants offered valuable perspectives on the tool as bystanders who have expertise in the feedback process. They shared many of the positive sentiments expressed by student participants but also reported unexpected challenges around the implementation of the tool, some of which were contradictory to student experiences. They indicated that the tool threatened essential opportunities for verbal feedback, feeling that students viewed standalone written feedback as sufficient. They also expressed concerns about the disproportionate weighing of process-based feedback and worried that other forms of feedback, task-based and individual-based, would fall out of practice [15]. One tutor participant felt that the tool excluded them from the feedback process:

“I usually would ask at the end of each group, okay, how's group process? How do you think you're doing in the process? And then how am I doing and what would you like me to do differently to facilitate? I didn't do that at all this MF just (be)cause there wasn't really space for it.” (Tutor Group)

The anonymous nature of the tool was repeatedly raised as a strength and a weakness. Tutor participants expressed concerns about the potential threat posed to them by anonymity:

“I thought…the students would be taking this opportunity to make their feelings about the tutorial and in particular the tutor. But there wasn't one, at least I didn't see one…if the students all say terrible things about me…what I'm I supposed to do about it?” (Tutor Group)

With the fear of negative comments arising and a feeling of exclusion from the tool’s feedback process, tutors may experience a threat to their roles with its implementation. In contrast, student participants viewed anonymity as an opportunity:

“Anonymity is probably one of the biggest strengths of the tool, just because there are potentially situations where somebody doesn't feel safe or comfortable being able to share something about somebody else.” (Group B)

Acknowledging the presence of dominant and non-dominant group members, students were excited by the prospect of the tool inviting perspectives that may otherwise be silenced. However, they also acknowledged the false sense of anonymity posed by the tool. Written feedback necessitates a verbal debriefing, meaning that anonymous reporters would inevitably need to identify themselves and elaborate on their comments. The disagreement around the tool’s anonymity, both between and within student and tutor participants, permits space for compromise and mitigation of the threat posed to tutors.

## Discussion

We found that the use of a group function tool to facilitate feedback conversations within PBL addressed many of the problems posed in the peer feedback literature. The tool provided a starting point for students to engage in feedback; it provided them with a benchmark for the attributes of well-functioning groups and informal teaching around feedback content. It provided much-needed objectivity to the feedback process and held groups accountable for the goals they set for themselves. However, the operationalization of the tool made explicit some tensions around the feedback process, reinforcing the literature’s consensus that variation is needed.

What should feedback look like? The collective bias of students and researchers was that feedback is for coaching, but this is evidently not always true. Indeed, this sentiment is reinforced by existing research that shows the ideal ratio of positive to constructive feedback is 5.6 to 1 [27]. Groups struggled between feeling that appreciative feedback was helpful and that it was counter-productive to forward movement, but appreciative feedback is known to promote confidence and personal growth in low-performing students [9]. Students should be careful not to downplay the value of appreciative feedback when using the tool.

Should structure be strictly applied? Groups carried an assumption that when structure is offered, it must be strictly applied. Consequently, they struggled with both applying structure that was needed and inviting flexibility in the tool’s implementation. As per the Human Factors Framework, the usability of a tool requires its properties to align with the user’s understanding of usefulness [24]. This is an inherently subjective appraisal, so groups must apply the tool in a way that maximizes its usability based on their own perceptions of usability. Thus, while structure is an essential characteristic of the tool, students will need to determine the appropriate level of structure based on their preferences.

Is verbal better than written feedback? Participants could not definitively agree on whether the written nature of the tool helped or harmed their process. Given the breadth of advantages and disadvantages posed, striking a balance is evidently the best way to integrate the tool into the feedback process. Indeed, auditory and written information are known to be processed distinctly from one another [28,29]. As such, balancing two modalities of feedback provides two opportunities to convey feedback, increasing the likelihood of impact. Groups will need to reflect on their needs and preferences to decide on an appropriate division of written and verbal feedback.

How much time is too much for feedback? Groups seek to minimize time spent on feedback and belabour the increased time demand of the tool, but they simultaneously underscore its centrality to the PBL process. Students should view feedback as a part of the learning process, not in conflict with it. They should take more or less time depending on their needs at any given time. For example, more experienced groups can devote less time to feedback, but groups experiencing plateaus in their group function, experienced or not, require additional time for feedback [14].

Does anonymity remove barriers or create risks? There was no consensus on whether anonymity is an advantage of the tool or even whether the tool offers real anonymity. During the development of the tool, anonymity arose as a clear advantage, but new problems were raised [21]. Accordingly, the literature reinforces potential risks of purely anonymous feedback, especially for tutors [30]. To mitigate the potential risks of anonymity, students should leverage it when needed, but not let it interfere with the inherently conversational nature of a feedback discussion.

Our findings provide a guide to implement the tool in PBL feedback processes and underscores the need to exercise caution in being too prescriptive. Instructions should be offered to structure the tool, but flexibility should be emphasized. Peer feedback is intrinsically self-directed, and the tool must preserve this. Table 2 summarizes the recommendations for implementing the tool. We recommend pairing the tool with some examples of how students may choose to implement it in their own groups. For example, using it before or after a verbal discussion, completing all sections or only predetermined sections, or using it independently or collaboratively. Groups could apply these instructions in a choose-your-own-adventure manner. The instructions should alert groups to the tensions identified in our results and prompt them to manage them according to their specific needs. Finally, the tool’s use should develop over time and may be tailored to meet the needs of students and tutors with varying levels of PBL and peer feedback experience.

**Table 2:** Recommendations for the implementation of the group function reflection tool

Theme	Recommendation
Types of feedback	Allow space for ample appreciative feedback
Feedback structure	Provide a range of examples of how groups could implement the tool and allow them to choose and augment as desired
Written and verbal feedback	Use both modalities and transition to favour one over the other as groups establish their preferences
Time	Gradually decrease time spent on feedback as the group progresses, leaving space to re-increase time as need arises
Anonymity	Conduct anonymous feedback when barriers to open discussion are suspected but prioritize open conversation where possible

Some limitations of this study and their implications on its transferability should be discussed. Introduction of the tool into the feedback process inevitably changes the nature of feedback itself. Therefore, while our findings offer interesting hints into the feedback behaviours of PBL groups more broadly, we cannot generalize these findings to describe PBL feedback behaviour outside of the context of the tool. We recommend additional investigation to assess whether these tensions are present in more organic PBL feedback processes. Additionally, this study was conducted in the setting of an academic program which emphasizes and values PBL and peer feedback. As a result, the feedback knowledge and skill level of student and tutor participants and perceived value of the tool may be overestimated. We encourage additional investigation to understand the implementation of this tool in programs with alternative curricula (for example, challenge-based learning, team-based learning, or didactic pedagogies). Finally, given that PBL group size is consistent across participating groups, it was not possible to assess the potential impact of group size on feedback behaviour.

## Conclusions

Peer feedback is an essential part of the PBL process, but there is little guidance on how to effectively structure these processes and students struggle without effective scaffolding. A newly developed group function reflection tool provides an opportunity to structure the PBL peer feedback process, and this proved to be beneficial to PBL groups. However, its implementation exposed tensions around feedback, which emphasize the need for flexibility in the application of scaffolds. For the tool to be successfully implemented, educators should alert tutors and students to the potential conflicts associated with the tool and provide suggestions for its use but allow groups to apply it in a flexible and self-directed manner. Future research should be conducted on the tool's implementation in non-PBL contexts or PBL contexts with different group sizes and compositions. Future research should also assess the generalizability of feedback behaviours with the tool in more organic PBL feedback processes.
